# Regression of syndesmophyte after bone marrow transplantation for acute myeloid leukemia in a patient with ankylosing spondylitis: a case report

**DOI:** 10.1186/1752-1947-6-250

**Published:** 2012-08-21

**Authors:** Hae Kyung Yang, Su-Jin Moon, Jae Ho Shin, Seung-Ki Kwok, Kyung Su Park, Sung-Hwan Park, Ho-Youn Kim, Ji Hyeon Ju

**Affiliations:** 1 Division of Rheumatology, Department of Internal Medicine, Seoul St. Mary’s Hospital, College of Medicine, Catholic Institute of Medical Science, The Catholic University of Korea, 505 Banpo-dong, Seocho-gu, Seoul, South Korea; 2 Division of Rheumatology, Department of Internal Medicine, Seoul St. Mary’s Hospital, College of Medicine, Catholic Institute of Medical Science, The Catholic University of Korea, Wonmi-gu, Bucheon, Gyeonggi-do, South Korea

## Abstract

**Introduction:**

Disease progression of ankylosing spondylitis has been considered irreversible. However, we report a case of spontaneous regression of syndesmophyte development following allogeneic peripheral blood stem cell transplantation in a patient with acute myeloid leukemia, who was also diagnosed as having ankylosing spondylitis. To the best of our knowledge, this is the first case report presenting the partial radiologic regression of syndesmophytes.

**Case presentation:**

A 39-year-old man with active ankylosing spondylitis achieved clinical remission and partial radiological regression of cervical spine syndesmophytes following a peripheral blood stem cell transplantation for acute myeloid leukemia. Our patient received an allogeneic peripheral blood stem cell transplantation following a pre-transplantation conditioning regimen of total body irradiation and cyclophosphamide. The donor was a human leukocyte antigen-matched 29-year-old man. Our patient has remained asymptomatic and has received no medication for ankylosing spondylitis for nearly three years.

**Conclusions:**

Several explanations are proposed for the regression of syndesmophytes and clinical improvement in active ankylosing spondylitis observed in our patient, including changes in bone remodeling and immune reconstitution following stem cell transplantation, the effect of immunosuppressive agents, or fluctuation in the natural course of ankylosing spondylitis although further studies are required. The regression of syndesmophytes in ankylosing spondylitis in this case raises the possibility that stem cell transplantation might contribute to the development of a novel therapeutic strategy for treatment of the disease.

## Introduction

Ankylosing spondylitis, an inflammatory rheumatologic disease that affects the axial skeleton resulting in characteristic inflammatory back pain, is uniquely associated with the major histocompatibility complex class I molecule, human leukocyte antigen (HLA)-B27 [1]. The current goals for treatment of this disease involve efforts to improve quality of life through pain control and alleviation of inflammation, prevent progressive structural damage, and preserve motor function [2]. Therapeutic modalities include non-pharmacological treatment such as regular exercise, and pharmacological treatment including non-steroidal anti-inflammatory drugs (NSAIDs), analgesics, glucocorticoids, disease-modifying anti-rheumatic drugs, and anti-tumor necrosis factor (TNF) therapy. Surgical treatment may be considered for severely disabling deformities. One of the hallmarks of ankylosing spondylitis is the formation of vertebral syndesmophytes, which may lead to diminished physical function and restricted spinal motion [3]. Disease progression and structural damage have been considered irreversible; however, we report a case of spontaneous regression of syndesmophyte development following allogeneic peripheral blood stem cell transplantation in a patient with acute myeloid leukemia, who was also diagnosed as having ankylosing spondylitis.

## Case presentation

A 39-year-old man was admitted to the hematologic department of our facility for peripheral blood stem cell transplantation in April 2009, following an initial diagnosis of acute myeloid leukemia in October 2008. An initial complete remission was achieved with induction chemotherapy and two cycles of consolidation chemotherapy with an IDV/BHAC (idarubicin-N4-behenoyl-L-β-D arabinofuranosyl-cytosine) regimen. Prior to the stem cell transplantation, our patient was referred to a rheumatologist for consultation regarding progressive neck pain and stiffness. He had been diagnosed as having ankylosing spondylitis 20 years previously, but never received regular clinical treatment or follow-up. Additionally, he was also not receiving regular medication for ankylosing spondylitis, but was instead using painkillers such as nonsteroidal anti-inflammatory drugs (NSAIDs) or intermittent physical rehabilitation to alleviate the progressively worsening neck pain. A physical examination of our patient revealed limited motion of the cervical spine, although his chest expansion and the range of motion of the lumbar spine were within normal limits. The peripheral blood counts reported were as follows: hemoglobin, 10.6g/dL; white blood cell count, 4720 × 10^9^ cells/L with normal differential cell count; and platelets, 128 × 10^9^ cells/L. His erythrocyte sedimentation rate was 66mm/hour and his C-reactive protein level was 0.29mg/dL. The results of immunological studies were negative for anti-nuclear antibody and both complement and immunoglobulin levels were within normal ranges, although HLA-B27 was positive. Urine analysis results were unremarkable. Radiologic findings were consistent with ankylosing spondylitis: bilateral sacroiliac joint fusion and bamboo spine. Examination of the cervical spine revealed C5 to C6 disc space narrowing with the formation of syndesmophytes.

Our patient was initially treated with NSAIDs and was scheduled to receive anti-TNF therapy following stem cell transplantation. In April 2009, he received allogeneic peripheral blood stem cell transplantation following a pre-transplantation conditioning regimen of 1320cGy total body irradiation and 60mg/kg cyclophosphamide for two days. The donor was an HLA-matched 29-year-old man. The HLA phenotype of our patient was A 1102, 3303; B 2704, 5801; C 0302, 1202 and that of the donor was the same. Our patient received an intravenous injection of 0.03mg/kg tacrolimus following oral administration of 0.120mg/kg tacrolimus, and intravenous injection of 5mg/body surface area methotrexate for graft-versus-host disease (GVHD) prophylaxis. No evidence of acute GVHD was reported following stem cell transplantation. Our patient was discharged one month following the stem cell transplantation without any complications.

**Figure 1:**
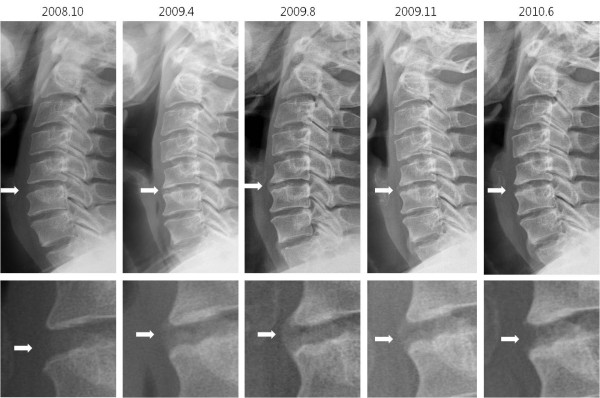
**Cervical spine X-rays.** Serial X-rays of the cervical spine revealed syndesmophyte formation between C5 and C6 and subsequent regression following peripheral blood stem cell transplantation in April 2008.

**Figure 2:**
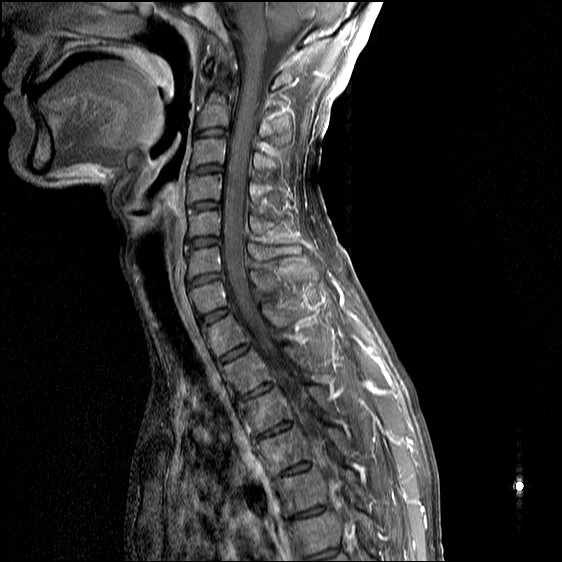
**Magnetic resonance imaging scan of the spine from May 2011.** The magnetic resonance imaging scan acquired in May 2011 indicated no evidence of osteitis in the cervical spine.

Five months after the peripheral blood stem cell transplantation, our patient reported reduced posterior neck pain, and an X-ray of the cervical spine revealed partial regression of the syndesmophytes (Figure 1). A magnetic resonance imaging (MRI) scan of our patient’s spine 25 months after the peripheral blood stem cell transplantation indicated no osteitis in the cervical spine (Figure 2). Furthermore, neither anti-TNF therapy or NSAID medication were prescribed due to our patient’s improved condition. Our patient has remained asymptomatic and has received no medication for ankylosing spondylitis for nearly three years. He received immunosuppressive therapy with tacrolimus for seven months following the peripheral blood stem cell transplantation, but all medication was stopped after that period. His condition has been stable without any recurrence of the hematologic disease.

## Discussion

To the best of our knowledge, this is the first case report presenting partial radiologic regression of syndesmophytes in a patient with ankylosing spondylitis. In our patient’s case, an individual positive for HLA-B2704 received a peripheral blood stem cell transplantation from an unrelated fully matched donor who was also positive for HLA-B2704. It is not clear whether and how the immunological change following the peripheral blood stem cell transplantation induced spontaneous regression of the syndesmophytes.

Several previous studies have reported feasible associations between hematologic malignancies, stem cell transplantation, and rheumatologic disease. Jantunen *et al*. reported complete clinical remission in active ankylosing spondylitis following chemotherapy and autologous stem cell transplantation for lymphoma [4]. However, the development of rheumatologic manifestations, such as arthralgias and arthritis, have been reported following allogeneic bone marrow transplantation or peripheral blood stem cell transplantation [5]. Some cases of hematological malignancies were preferentially expressed in HLA-B27 carriers [6]. Hematopoietic stem cell transplantation and resetting the immune system for severe autoimmune disease have proven to be effective forms of treatment in some cases [7].

Several explanations are proposed for the regression of syndesmophytes and clinical improvement in active ankylosing spondylitis observed in our patient, including changes in bone remodeling and immune reconstitution following stem cell transplantation, the effect of immunosuppressive agents, or fluctuation in the natural course of ankylosing spondylitis although further studies are required. Comparison of MRI scan results acquired before and after peripheral blood stem cell transplantation would provide more information about changes in bony structure, but pre-transplant images were not available for our patient. The partial regression of syndesmophytes in ankylosing spondylitis in this case raises the possibility that stem cell transplantation might contribute to the development of a novel therapeutic strategy for treatment of the disease.

## Conclusions

We report a case of partial radiologic regression of syndesmophytes in a patient with ankylosing spondylitis. Our case raises the possibility that stem cell transplantation may contribute to the development of a novel therapeutic strategy for this disease.

## Consent

Written informed consent was obtained from the patient for publication of this case report and any accompanying images. A copy of the written consent is available for review by the Editor-in-Chief of this journal.

## Competing interests

The authors declare that they have no competing interests. This research was partially supported by Basic Science Research Program through the National Research Foundation of Korea (NRF) funded by the Ministry of Education, Science and Technology (331-2008-1-E00144).

## Authors’ contributions

HKY and SJM were the main authors and performed the clinical assessment and follow-up. JHS and SKK performed the clinical assessment and bibliographic research. KSP, SHP and HYK were major contributors in the writing the manuscript. JHJ performed the clinical assessment and bibliographic research as a corresponding author. All authors have read and approved the final manuscript.

## References

[B1] Braun J Sieper J Ankylosing spondylitis Lancet 2007 369 1379 1390 10.1016/S0140-6736(07)60635-7 17448825

[B2] Braun J van den Berg R Baraliakos X Boehm H Burgos-Vargas R Collantes-Estevez E Dagfinrud H Dijkmans B Dougados M Emery P Geher P Hammoudeh M Inman RD Jongkees M Khan MA Kiltz U Kvien T Leirisalo-Repo M Maksymowych WP Olivieri I Pavelka K Sieper J Stanislawska-Biernat E Wendling D Ozgocmen S van Drogen C van Royen B van der Heijde D 2010 update of the ASAS/EULAR recommendations for the management of ankylosing spondylitis Ann Rheum Dis 2011 70 896 904 10.1136/ard.2011.151027 21540199 PMC3086052

[B3] Landewé R Dougados M Mielants H van der Tempel H van der Heijde D Physical function in ankylosing spondylitis is independently determined by both disease activity and radiographic damage of the spine Ann Rheum Dis 2009 68 863 867 10.1136/ard.2008.091793 18628283

[B4] Jantunen E Myllykangas-Luosujarvi R Kaipiainen-Seppanen O Nousiainen T Autologous stem cell transplantation in a lymphoma patient with a long history of ankylosing spondylitis Rheumatology (Oxford) 2000 39 563 564 10.1093/rheumatology/39.5.563 10852991

[B5] Koch B Kranzhofer N Pfreundschu M Pees HW Trumper L First manifestations of seronegative spondylarthropathy following autologous stem cell transplantation in HLA-B27-positive patients Bone Marrow Transplant 2000 26 673 675 10.1038/sj.bmt.1702565 11035374

[B6] Au WY Hawkins BR Cheng N Lie AK Liang R Kwong YL Risk of haematological malignancies in HLA-B27 carriers Br J Haematol 2001 115 320 322 10.1046/j.1365-2141.2001.03114.x 11703328

[B7] Tyndall A Gratwohl A Adult stem cell transplantation in autoimmune disease Curr Opin Hematol 2009 16 285 291 10.1097/MOH.0b013e32832aacb3 19465851

